# *N*,*N*′,*N*′′,*N*′′′-Tetrakis(2-methylphenyl)­oxybis(phospho­nic diamide): a redetermination at 150 K with Mo *K*α radiation

**DOI:** 10.1107/S1600536811002091

**Published:** 2011-01-22

**Authors:** Mehrdad Pourayoubi, Zdeňka Padělková, Mahnaz Rostami Chaijan, Aleš Růžička

**Affiliations:** a Department of Chemistry, Ferdowsi University of Mashhad, Mashhad, 91779, Iran; b Department of General and Inorganic Chemistry, Faculty of Chemical Technology, University of Pardubice, Studentská 573, Pardubice 532 10, Czech Republic

## Abstract

The structure of the title compound, C_28_H_32_N_4_O_3_P_2_, has been redetermined at 150 K, with much improved precision. The structure and mol­ecular packing of the title compound was previously determined using Cu *K*α radiation, with an *R* value of 0.0933 [Cameron *et al.* (1978 ▶). *Z. Naturforsch. Teil B*, **33**, 728–730]. The *c*-axis length in this structure [13.8401 (8) Å] is almost half that reported in the original study. In the title compound, two (C_6_H_4_(2-CH_3_)NH)_2_P(O) units are bridged *via* an O atom [P—O—P = 133.31 (11)°]. The P atoms adopt a slightly distorted tetra­hedral coordination geometry. In the crystal, mol­ecules are linked *via* N—H⋯OP hydrogen bonds into extended chains parallel to the *c* axis. An intra­molecular N—H⋯O=P hydrogen bond is also found in the mol­ecule.

## Related literature

For the previous determination of this structure, see: Cameron *et al.* (1978 ▶). For bond lengths and angles in related structures, see: Pourayoubi *et al.* (2010 ▶); Sabbaghi *et al.* (2010 ▶). 
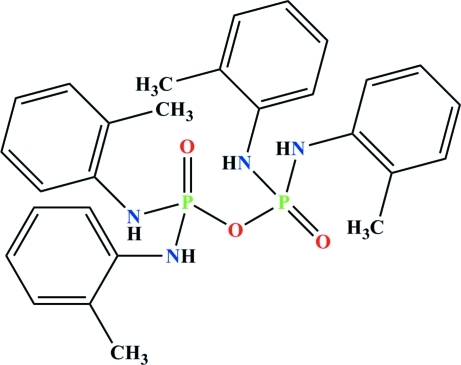


## Experimental

### 

#### Crystal data

C_28_H_32_N_4_O_3_P_2_
*M* *_r_* = 534.52
Monoclinic, 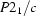

*a* = 14.2621 (6) Å
*b* = 15.7029 (11) Å
*c* = 13.8401 (8) Å
β = 118.915 (4)°
*V* = 2713.2 (3) Å^3^
*Z* = 4
Mo *K*α radiation
μ = 0.20 mm^−1^
*T* = 150 K
0.45 × 0.33 × 0.21 mm

#### Data collection

Bruker–Nonius KappaCCD area-detector diffractometer
Absorption correction: Gaussian integration (Coppens & Hamilton, 1970 ▶) *T*_min_ = 0.942, *T*_max_ = 0.969
19090 measured reflections
6133 independent reflections
4719 reflections with *I* > 2σ(*I*)
*R*_int_ = 0.047

#### Refinement

*R*[*F* ^2^ > 2σ(*F* ^2^)] = 0.054
*wR*(*F* ^2^) = 0.121
*S* = 1.18
6133 reflections
334 parameters
H-atom parameters constrained
Δρ_max_ = 0.38 e Å^−3^
Δρ_min_ = −0.51 e Å^−3^

### 

Data collection: *COLLECT* (Hooft, 1998 ▶) and *DENZO* (Otwinowski & Minor, 1997 ▶); cell refinement: *COLLECT* and *DENZO*; data reduction: *COLLECT* and *DENZO*; program(s) used to solve structure: *SIR92* (Altomare *et al.*, 1994 ▶); program(s) used to refine structure: *SHELXL97* (Sheldrick, 2008 ▶); molecular graphics: *ORTEP-3* (Farrugia, 1997 ▶) and *Mercury* (Macrae *et al.*, 2008 ▶); software used to prepare material for publication: *SHELXL97*.

## Supplementary Material

Crystal structure: contains datablocks I, global. DOI: 10.1107/S1600536811002091/sj5092sup1.cif

Structure factors: contains datablocks I. DOI: 10.1107/S1600536811002091/sj5092Isup2.hkl

Additional supplementary materials:  crystallographic information; 3D view; checkCIF report

## Figures and Tables

**Table 1:** Hydrogen-bond geometry (Å, °)

*D*—H⋯*A*	*D*—H	H⋯*A*	*D*⋯*A*	*D*—H⋯*A*
N111—H111⋯O3^i^	0.86	2.16	2.926 (2)	149
N112—H112⋯O3	0.86	2.16	2.906 (2)	144
N113—H113⋯O2^ii^	0.86	1.97	2.814 (2)	167

Symmetry codes: (i) 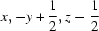; (ii) 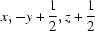.

**Fig. 1.**
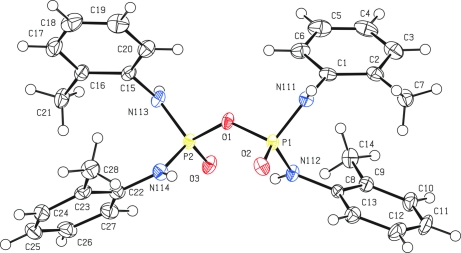
Molecular structure and atom labeling scheme for title compound with displacement ellipsoids at the 50% probability level.

**Fig. 2.**
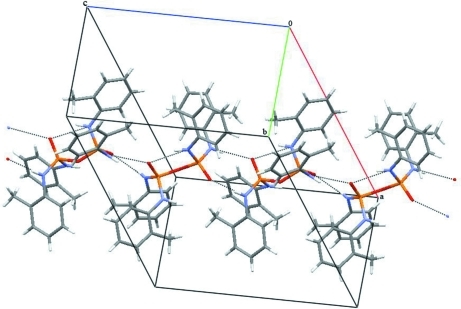
A view of H-bonded chain in the crystal network of title compound. N–H···OP hydrogen bonds are shown as dashed lines.

## supplementary crystallographic information

### Comment

The structure and molecular packing of *µ*-oxo-bis(phosphenyl-*o*-toluidide) was previously reported at ambient temperature by Cameron *et al.* (1978) using 2595 independent reflections significantly above the background with Cu *K*α radiation; *R* = 0.0933. The unit-cell length *c* and density were respectively reported as 25.26 (3) Å and 1.255 g cm^-3^.

Here, we report on the low temperature X-ray determination of the title compound (Fig. 1) at 150 K using Mo *K*α radiation, with some improved precision. The unit-cell length *c* (of 13.8401 (8) Å) and density (of 1.309 g cm^-3^) in this structure are very different from the previously reported values.

In the title compound, two (C_6_H_4_(2-CH_3_)NH)_2_P(O) moieties are bridged *via* an oxygen atom (P1—O1—P2 angle = 133.31 (11)°); the P1—O1 and P2—O1 bond lengths of 1.6014 (16) and 1.6017 (16) Å are standard for the P—O—P moiety (Pourayoubi *et al.*, 2010). The P atoms adopt a slightly distorted tetrahedral environment. The bond angles around the P atoms are in the range of 101.05 (9)° to 116.62 (11)° for P1 and 100.12 (10)° to 117.97 (11)° for P2.

The P1—O2 and P2—O3 bond lengths (1.4681 (17) and 1.4736 (17) Å) and the P—N bond lengths (1.630 (2), 1.6376 (19), 1.612 (2) and 1.634 (2) Å) are standard for this type of compound; for example in [4-H_3_C—C_6_H_4_O]P(O)[NHC_6_H_4_-2-CH_3_]_2_ (Sabbaghi *et al.*, 2010), P═O = 1.4692 (12) Å and P—N = 1.6268 (15) and 1.6279 (15) Å).

An intramolecular N–H···OP hydrogen bond (N···O = 2.906 (2) Å) is found between the oxygen atom of the P2 phosphoryl group and the N–H hydrogen atom of one of the amide moieties linked to the P1 atom. In the crystal structure, molecules are linked *via* N–H···OP hydrogen bonds (Fig. 2) into extended chains parallel to the *c* axis (N···O = 2.814 (2) & 2.926 (2) Å).

### Experimental

To a solution of (11.42 mmol) phosphoryl chloride in chloroform (10 ml), a solution of *o*-toluidine (68.52 mmol) in chloroform (10 ml) was added dropwise at 268 K. After 4 h, the solvent was removed in vacuum. Single crystals were obtained from a mixture of chloroform/n-heptane after slow evaporation at room temperature. ^31^P{^1^H} NMR (202.45 MHz, DMSO-d6, 300.0 K, H_3_PO_4_ external): -5.01 p.p.m. (*s*). ^1^H NMR (500.13 MHz, DMSO-d6, 300.0 K, TMS): 2.03 (*s*, 12H, 4CH_3_), 6.87–7.06 (*m*, 12H, Ar—H), 7.29 (*m*, 4H, Ar—H), 7.35 p.p.m. (*d*, ^2^J(P,H) = 7.9 Hz, 4H, NH). IR (KBr, cm^-1^): 3746.3, 3413.2, 3300.4, 3188.5, 2927.3, 2854.8, 2358.8, 1675.8, 1599.4, 1496.1, 1402.5, 1226.5, 1111.8, 980.2, 844.9, 750.9.

### Refinement

All the H atoms were discernible in the difference electron density map. However, all the H atoms were positioned geometrically and refined as riding on their parent C or N atoms, with N—H = 0.86 Å, C—H = 0.98 Å for methyl, C—H = 0.93 Å for aromatic hydrogen atoms, U(H) = 1.2*U*_eq_(C/N) for the amine and U(H) = 1.5*U*_eq_(C) for methyl H atoms, respectively.

### Crystal data

**Table:**

C_28_H_32_N_4_O_3_P_2_	*Z* = 4
*M**_r_* = 534.52	*F*(000) = 1128
Monoclinic, *P*2_1_/*c*	*D*_x_ = 1.309 Mg m^−^^3^
Hall symbol: -P 2ybc	Mo *K*α radiation, λ = 0.71073 Å
*a* = 14.2621 (6) Å	θ = 1–27.5°
*b* = 15.7029 (11) Å	µ = 0.20 mm^−^^1^
*c* = 13.8401 (8) Å	*T* = 150 K
β = 118.915 (4)°	Needle, colourless
*V* = 2713.2 (3) Å^3^	0.45 × 0.33 × 0.21 mm

### Data collection

**Table:**

Bruker–Nonius KappaCCD area-detector diffractometer	6133 independent reflections
Radiation source: fine-focus sealed tube	4719 reflections with *I* > 2σ(*I*)
graphite	*R*_int_ = 0.047
Detector resolution: 9.091 pixels mm^-1^	θ_max_ = 27.5°, θ_min_ = 1.6°
φ and ω scans to fill the Ewald sphere	*h* = −17→18
Absorption correction: integration Gaussian integration (Coppens & Hamilton, 1970)	*k* = −20→18
*T*_min_ = 0.942, *T*_max_ = 0.969	*l* = −17→17
19090 measured reflections	

### Refinement

**Table:**

Refinement on *F*^2^	Primary atom site location: structure-invariant direct methods
Least-squares matrix: full	Secondary atom site location: difference Fourier map
*R*[*F*^2^ > 2σ(*F*^2^)] = 0.054	Hydrogen site location: inferred from neighbouring sites
*wR*(*F*^2^) = 0.121	H-atom parameters constrained
*S* = 1.18	*w* = 1/[σ^2^(*F*_o_^2^) + (0.026*P*)^2^ + 3.5968*P*] where *P* = (*F*_o_^2^ + 2*F*_c_^2^)/3
6133 reflections	(Δ/σ)_max_ < 0.001
334 parameters	Δρ_max_ = 0.38 e Å^−^^3^
0 restraints	Δρ_min_ = −0.51 e Å^−^^3^

### Special details

**Table:**

Geometry. All e.s.d.'s (except the e.s.d. in the dihedral angle between two l.s. planes) are estimated using the full covariance matrix. The cell e.s.d.'s are taken into account individually in the estimation of e.s.d.'s in distances, angles and torsion angles; correlations between e.s.d.'s in cell parameters are only used when they are defined by crystal symmetry. An approximate (isotropic) treatment of cell e.s.d.'s is used for estimating e.s.d.'s involving l.s. planes.
Refinement. Refinement of *F*^2^ against ALL reflections. The weighted *R*-factor *wR* and goodness of fit *S* are based on *F*^2^, conventional *R*-factors *R* are based on *F*, with *F* set to zero for negative *F*^2^. The threshold expression of *F*^2^ > σ(*F*^2^) is used only for calculating *R*-factors(gt) *etc*. and is not relevant to the choice of reflections for refinement. *R*-factors based on *F*^2^ are statistically about twice as large as those based on *F*, and *R*- factors based on ALL data will be even larger.

### Fractional atomic coordinates and isotropic or equivalent isotropic displacement parameters (Å^2^)

**Table:**

	*x*	*y*	*z*	*U*_iso_*/*U*_eq_	
P1	0.71234 (5)	0.23508 (4)	0.23550 (5)	0.01571 (14)	
P2	0.81104 (5)	0.21568 (4)	0.47636 (5)	0.01629 (14)	
O3	0.71315 (13)	0.18618 (12)	0.47678 (13)	0.0220 (4)	
O2	0.77228 (13)	0.18044 (11)	0.19840 (14)	0.0222 (4)	
N111	0.67768 (15)	0.32469 (13)	0.16770 (15)	0.0171 (4)	
H111	0.7047	0.3375	0.1258	0.021*	
N112	0.60850 (15)	0.19217 (13)	0.23720 (15)	0.0181 (4)	
H112	0.6126	0.1794	0.2995	0.022*	
O1	0.78382 (12)	0.26154 (11)	0.36239 (12)	0.0195 (4)	
N113	0.87451 (15)	0.28468 (13)	0.57247 (15)	0.0183 (4)	
H113	0.8426	0.3037	0.6073	0.022*	
N114	0.89635 (16)	0.14390 (13)	0.48176 (16)	0.0207 (4)	
H114	0.9035	0.1363	0.4242	0.025*	
C15	0.97931 (18)	0.31677 (16)	0.6050 (2)	0.0192 (5)	
C8	0.51193 (18)	0.17513 (15)	0.13812 (18)	0.0171 (5)	
C14	0.4100 (2)	0.25236 (18)	0.2194 (2)	0.0263 (6)	
H14A	0.3375	0.2691	0.1963	0.032*	
H14B	0.4543	0.3021	0.2366	0.032*	
H14C	0.4356	0.2169	0.2837	0.032*	
C10	0.3220 (2)	0.18702 (18)	0.0296 (2)	0.0268 (6)	
H10	0.2563	0.2049	0.0211	0.032*	
C27	0.9154 (2)	0.06277 (17)	0.6406 (2)	0.0259 (6)	
H27	0.8443	0.0744	0.6200	0.031*	
C23	1.0662 (2)	0.07556 (16)	0.6064 (2)	0.0231 (5)	
C1	0.60452 (19)	0.38319 (15)	0.1753 (2)	0.0193 (5)	
C2	0.5062 (2)	0.40163 (16)	0.0836 (2)	0.0226 (5)	
C9	0.41430 (19)	0.20379 (16)	0.12797 (19)	0.0206 (5)	
C24	1.1247 (2)	0.02480 (17)	0.6984 (2)	0.0294 (6)	
H24	1.1947	0.0104	0.7174	0.035*	
C21	1.0415 (2)	0.25452 (19)	0.7949 (2)	0.0297 (6)	
H21A	1.1022	0.2605	0.8673	0.036*	
H21B	1.0307	0.1954	0.7750	0.036*	
H21C	0.9789	0.2768	0.7950	0.036*	
C16	1.06130 (19)	0.30251 (16)	0.7133 (2)	0.0219 (5)	
C6	0.6332 (2)	0.42124 (17)	0.2774 (2)	0.0267 (6)	
H6	0.7003	0.4104	0.3374	0.032*	
C22	0.9598 (2)	0.09322 (16)	0.5770 (2)	0.0214 (5)	
C11	0.3249 (2)	0.1444 (2)	−0.0562 (2)	0.0326 (7)	
H11	0.2619	0.1348	−0.1218	0.039*	
C12	0.4213 (2)	0.11623 (19)	−0.0450 (2)	0.0305 (6)	
H12	0.4234	0.0873	−0.1025	0.037*	
C3	0.4368 (2)	0.45692 (18)	0.0985 (2)	0.0309 (6)	
H3	0.3708	0.4705	0.0386	0.037*	
C13	0.5154 (2)	0.13111 (17)	0.0530 (2)	0.0241 (5)	
H13	0.5806	0.1117	0.0615	0.029*	
C25	1.0816 (2)	−0.00486 (18)	0.7628 (2)	0.0327 (6)	
H25	1.1228	−0.0381	0.8247	0.039*	
C19	1.1003 (2)	0.39397 (19)	0.5620 (3)	0.0331 (6)	
H19	1.1132	0.4239	0.5116	0.040*	
C7	0.4739 (2)	0.36301 (18)	−0.0263 (2)	0.0264 (6)	
H7A	0.5312	0.3690	−0.0435	0.032*	
H7B	0.4112	0.3914	−0.0816	0.032*	
H7C	0.4585	0.3037	−0.0247	0.032*	
C20	0.9989 (2)	0.36202 (18)	0.5309 (2)	0.0261 (6)	
H20	0.9437	0.3714	0.4595	0.031*	
C26	0.9776 (2)	0.01502 (17)	0.7348 (2)	0.0300 (6)	
H26	0.9489	−0.0035	0.7788	0.036*	
C28	1.1164 (2)	0.11288 (19)	0.5422 (2)	0.0308 (6)	
H28A	1.1902	0.0960	0.5754	0.037*	
H28B	1.1120	0.1739	0.5429	0.037*	
H28C	1.0791	0.0928	0.4674	0.037*	
C18	1.1820 (2)	0.3807 (2)	0.6680 (3)	0.0354 (7)	
H18	1.2501	0.4021	0.6897	0.043*	
C5	0.5626 (3)	0.47486 (18)	0.2898 (3)	0.0347 (7)	
H5	0.5812	0.4991	0.3581	0.042*	
C17	1.1622 (2)	0.33534 (19)	0.7422 (2)	0.0314 (6)	
H17	1.2181	0.3266	0.8133	0.038*	
C4	0.4648 (2)	0.49159 (18)	0.2005 (3)	0.0344 (7)	
H4	0.4168	0.5270	0.2087	0.041*	

### Atomic displacement parameters (Å^2^)

**Table:**

	*U*^11^	*U*^22^	*U*^33^	*U*^12^	*U*^13^	*U*^23^
P1	0.0135 (3)	0.0217 (3)	0.0119 (3)	0.0002 (2)	0.0062 (2)	0.0018 (2)
P2	0.0149 (3)	0.0225 (3)	0.0113 (3)	−0.0025 (2)	0.0062 (2)	0.0001 (2)
O3	0.0189 (8)	0.0333 (10)	0.0141 (8)	−0.0056 (8)	0.0083 (7)	−0.0009 (7)
O2	0.0214 (8)	0.0264 (9)	0.0232 (9)	0.0059 (7)	0.0144 (7)	0.0043 (7)
N111	0.0195 (9)	0.0215 (10)	0.0146 (9)	−0.0007 (8)	0.0115 (8)	0.0009 (8)
N112	0.0163 (9)	0.0267 (11)	0.0119 (9)	−0.0035 (8)	0.0073 (8)	0.0021 (8)
O1	0.0163 (8)	0.0280 (9)	0.0113 (7)	−0.0049 (7)	0.0045 (6)	0.0020 (7)
N113	0.0150 (9)	0.0261 (11)	0.0150 (9)	−0.0024 (8)	0.0081 (8)	−0.0046 (8)
N114	0.0260 (11)	0.0239 (11)	0.0148 (9)	0.0019 (9)	0.0119 (8)	0.0005 (8)
C15	0.0151 (11)	0.0206 (12)	0.0221 (12)	−0.0010 (10)	0.0090 (9)	−0.0053 (10)
C8	0.0178 (11)	0.0167 (11)	0.0140 (10)	−0.0019 (9)	0.0055 (9)	0.0029 (9)
C14	0.0216 (12)	0.0355 (15)	0.0246 (13)	0.0017 (11)	0.0134 (10)	0.0018 (11)
C10	0.0167 (12)	0.0343 (15)	0.0249 (13)	−0.0031 (11)	0.0063 (10)	0.0033 (11)
C27	0.0308 (14)	0.0234 (13)	0.0265 (13)	0.0003 (11)	0.0163 (11)	0.0009 (11)
C23	0.0253 (13)	0.0198 (13)	0.0237 (12)	−0.0030 (10)	0.0115 (11)	−0.0085 (10)
C1	0.0245 (12)	0.0156 (11)	0.0247 (12)	−0.0012 (10)	0.0172 (10)	0.0011 (10)
C2	0.0249 (12)	0.0182 (12)	0.0280 (13)	0.0002 (10)	0.0154 (11)	0.0044 (10)
C9	0.0206 (12)	0.0228 (13)	0.0182 (11)	−0.0031 (10)	0.0092 (10)	0.0030 (10)
C24	0.0285 (14)	0.0237 (14)	0.0276 (14)	0.0045 (11)	0.0068 (11)	−0.0027 (11)
C21	0.0266 (13)	0.0372 (16)	0.0179 (12)	−0.0018 (12)	0.0049 (10)	−0.0012 (11)
C16	0.0172 (11)	0.0228 (13)	0.0227 (12)	0.0026 (10)	0.0072 (10)	−0.0052 (10)
C6	0.0338 (14)	0.0227 (13)	0.0274 (13)	−0.0032 (12)	0.0178 (12)	−0.0040 (11)
C22	0.0265 (13)	0.0183 (12)	0.0184 (11)	−0.0008 (10)	0.0101 (10)	−0.0029 (10)
C11	0.0237 (13)	0.0411 (17)	0.0192 (12)	−0.0111 (13)	−0.0007 (10)	−0.0013 (12)
C12	0.0367 (15)	0.0334 (15)	0.0184 (12)	−0.0098 (13)	0.0109 (11)	−0.0079 (11)
C3	0.0314 (14)	0.0262 (15)	0.0383 (15)	0.0041 (12)	0.0193 (13)	0.0078 (12)
C13	0.0246 (13)	0.0260 (14)	0.0210 (12)	−0.0015 (11)	0.0104 (10)	−0.0014 (10)
C25	0.0423 (16)	0.0217 (14)	0.0250 (13)	0.0037 (12)	0.0090 (12)	0.0032 (11)
C19	0.0330 (15)	0.0341 (16)	0.0426 (16)	−0.0031 (13)	0.0264 (14)	0.0005 (13)
C7	0.0255 (13)	0.0281 (14)	0.0241 (13)	0.0016 (11)	0.0107 (11)	0.0038 (11)
C20	0.0244 (13)	0.0295 (14)	0.0257 (13)	−0.0042 (11)	0.0130 (11)	−0.0025 (11)
C26	0.0429 (16)	0.0222 (13)	0.0267 (14)	−0.0017 (12)	0.0182 (13)	0.0024 (11)
C28	0.0271 (14)	0.0330 (15)	0.0343 (15)	−0.0002 (12)	0.0164 (12)	−0.0020 (12)
C18	0.0222 (13)	0.0353 (16)	0.0519 (18)	−0.0066 (12)	0.0203 (13)	−0.0101 (14)
C5	0.0517 (18)	0.0266 (15)	0.0364 (16)	−0.0027 (14)	0.0297 (15)	−0.0062 (12)
C17	0.0177 (12)	0.0350 (16)	0.0341 (15)	0.0005 (12)	0.0066 (11)	−0.0063 (12)
C4	0.0455 (17)	0.0195 (14)	0.0502 (18)	0.0071 (13)	0.0327 (15)	0.0024 (13)

### Geometric parameters (Å, °)

**Table:**

P1—O2	1.4681 (17)	C2—C7	1.489 (4)
P1—O1	1.6014 (16)	C24—C25	1.385 (4)
P1—N111	1.630 (2)	C24—H24	0.9300
P1—N112	1.6376 (19)	C21—C16	1.494 (4)
P2—O3	1.4736 (17)	C21—H21A	0.9601
P2—O1	1.6017 (16)	C21—H21B	0.9599
P2—N113	1.612 (2)	C21—H21C	0.9601
P2—N114	1.634 (2)	C16—C17	1.392 (4)
N111—C1	1.432 (3)	C6—C5	1.385 (4)
N111—H111	0.8600	C6—H6	0.9300
N112—C8	1.422 (3)	C11—C12	1.379 (4)
N112—H112	0.8599	C11—H11	0.9299
N113—C15	1.428 (3)	C12—C13	1.391 (4)
N113—H113	0.8600	C12—H12	0.9300
N114—C22	1.427 (3)	C3—C4	1.380 (4)
N114—H114	0.8600	C3—H3	0.9299
C15—C20	1.383 (4)	C13—H13	0.9300
C15—C16	1.403 (3)	C25—C26	1.375 (4)
C8—C13	1.387 (3)	C25—H25	0.9300
C8—C9	1.404 (3)	C19—C18	1.379 (4)
C14—C9	1.504 (3)	C19—C20	1.388 (4)
C14—H14A	0.9600	C19—H19	0.9300
C14—H14B	0.9600	C7—H7A	0.9601
C14—H14C	0.9599	C7—H7B	0.9600
C10—C11	1.382 (4)	C7—H7C	0.9601
C10—C9	1.387 (3)	C20—H20	0.9301
C10—H10	0.9300	C26—H26	0.9300
C27—C26	1.389 (4)	C28—H28A	0.9601
C27—C22	1.393 (4)	C28—H28B	0.9600
C27—H27	0.9300	C28—H28C	0.9600
C23—C24	1.388 (4)	C18—C17	1.386 (4)
C23—C22	1.396 (3)	C18—H18	0.9300
C23—C28	1.504 (4)	C5—C4	1.369 (4)
C1—C2	1.393 (3)	C5—H5	0.9300
C1—C6	1.401 (3)	C17—H17	0.9300
C2—C3	1.404 (4)	C4—H4	0.9300
			
O2—P1—O1	111.45 (10)	C16—C21—H21C	109.4
O2—P1—N111	111.65 (10)	H21A—C21—H21C	109.5
O1—P1—N111	105.22 (10)	H21B—C21—H21C	109.5
O2—P1—N112	116.62 (11)	C17—C16—C15	117.3 (2)
O1—P1—N112	101.05 (9)	C17—C16—C21	121.1 (2)
N111—P1—N112	109.81 (10)	C15—C16—C21	121.6 (2)
O3—P2—O1	111.48 (9)	C5—C6—C1	120.6 (3)
O3—P2—N113	111.30 (10)	C5—C6—H6	119.7
O1—P2—N113	106.40 (10)	C1—C6—H6	119.7
O3—P2—N114	117.97 (11)	C27—C22—C23	120.6 (2)
O1—P2—N114	100.12 (10)	C27—C22—N114	119.8 (2)
N113—P2—N114	108.51 (11)	C23—C22—N114	119.6 (2)
C1—N111—P1	122.72 (15)	C12—C11—C10	120.1 (2)
C1—N111—H111	118.7	C12—C11—H11	119.9
P1—N111—H111	118.6	C10—C11—H11	120.0
C8—N112—P1	121.64 (15)	C11—C12—C13	119.7 (3)
C8—N112—H112	119.2	C11—C12—H12	120.2
P1—N112—H112	119.2	C13—C12—H12	120.2
P1—O1—P2	133.31 (11)	C4—C3—C2	121.2 (3)
C15—N113—P2	125.59 (16)	C4—C3—H3	119.3
C15—N113—H113	117.2	C2—C3—H3	119.5
P2—N113—H113	117.2	C8—C13—C12	119.9 (2)
C22—N114—P2	123.54 (17)	C8—C13—H13	120.0
C22—N114—H114	118.2	C12—C13—H13	120.1
P2—N114—H114	118.3	C26—C25—C24	119.7 (3)
C20—C15—C16	120.8 (2)	C26—C25—H25	120.2
C20—C15—N113	120.2 (2)	C24—C25—H25	120.1
C16—C15—N113	119.0 (2)	C18—C19—C20	119.5 (3)
C13—C8—C9	121.0 (2)	C18—C19—H19	120.2
C13—C8—N112	119.8 (2)	C20—C19—H19	120.3
C9—C8—N112	119.2 (2)	C2—C7—H7A	109.5
C9—C14—H14A	109.5	C2—C7—H7B	109.5
C9—C14—H14B	109.4	H7A—C7—H7B	109.5
H14A—C14—H14B	109.5	C2—C7—H7C	109.4
C9—C14—H14C	109.5	H7A—C7—H7C	109.5
H14A—C14—H14C	109.5	H7B—C7—H7C	109.5
H14B—C14—H14C	109.5	C15—C20—C19	120.6 (3)
C11—C10—C9	121.8 (2)	C15—C20—H20	119.8
C11—C10—H10	119.1	C19—C20—H20	119.5
C9—C10—H10	119.1	C25—C26—C27	120.0 (3)
C26—C27—C22	119.9 (3)	C25—C26—H26	120.0
C26—C27—H27	120.1	C27—C26—H26	120.0
C22—C27—H27	120.0	C23—C28—H28A	109.6
C24—C23—C22	117.9 (2)	C23—C28—H28B	109.3
C24—C23—C28	121.3 (2)	H28A—C28—H28B	109.5
C22—C23—C28	120.8 (2)	C23—C28—H28C	109.5
C2—C1—C6	120.3 (2)	H28A—C28—H28C	109.5
C2—C1—N111	121.0 (2)	H28B—C28—H28C	109.5
C6—C1—N111	118.7 (2)	C19—C18—C17	119.8 (3)
C1—C2—C3	117.8 (2)	C19—C18—H18	120.0
C1—C2—C7	121.6 (2)	C17—C18—H18	120.1
C3—C2—C7	120.6 (2)	C4—C5—C6	119.2 (3)
C10—C9—C8	117.5 (2)	C4—C5—H5	120.3
C10—C9—C14	121.1 (2)	C6—C5—H5	120.5
C8—C9—C14	121.3 (2)	C18—C17—C16	122.0 (3)
C25—C24—C23	121.8 (3)	C18—C17—H17	118.8
C25—C24—H24	119.2	C16—C17—H17	119.2
C23—C24—H24	119.0	C5—C4—C3	120.8 (3)
C16—C21—H21A	109.5	C5—C4—H4	119.5
C16—C21—H21B	109.4	C3—C4—H4	119.6
H21A—C21—H21B	109.5		

### Hydrogen-bond geometry (Å, °)

**Table:**

*D*—H···*A*	*D*—H	H···*A*	*D*···*A*	*D*—H···*A*
N111—H111···O3^i^	0.86	2.16	2.926 (2)	149
N112—H112···O3	0.86	2.16	2.906 (2)	144
N113—H113···O2^ii^	0.86	1.97	2.814 (2)	167

Symmetry codes: (i) *x*, −*y*+1/2, *z*−1/2; (ii) *x*, −*y*+1/2, *z*+1/2.
